# Lectures on Diseases of the Skin

**Published:** 1886-06

**Authors:** Henry Wile

**Affiliations:** Lecturer on Dermatology, Atlanta, Ga.


### LECTURES ON DISEASES OF THE SKIN.

DELIVERED AT THE ATLANTA MEDICAL COLLEGE DURING THE SESSION OF 1885–6, BY HENRY WILE, M. D., LECTURER ON DERMATOLOGY, ATLANTA, GA.

### LECTURE IV.

GENERAL PATHOLOGY—HYPERÆMIA, ACTIVE, PASSIVE—INFLAMMATION—DEGENERATIONS—HEMORRHAGE—HYPERTROPHY—ATROPHY.

Reported Stenographically for the ATLANTA MEDICAL AND SURGICAL JOURNAL by W. Kay Tewksbury.

On account of its position, the skin is superior to other organs of the body in the facility it offers for the study of pathological processes during life. Disease may be followed step by step through the different stages of development; all the physical signs may be appreciated, and slight transient changes can be observed with minuteness and detail. The importance of such *intra vitam* study of disease cannot be over-estimated for the light it sheds upon the pathology, not only of the skin, but of the other organs. There are many processes which take place in the tissues of the skin, which, though clearly defined and well marked during life, are not appreciated after death. This is doubtless the case with other organs of the body, and, as a great pathologist says, "it is easy to see that in many cases the result of the autopsy is not commensurate with the symptoms observed during life."

In diseases of the skin, except where profound alterations of structure have taken place, it therefore sometimes happens that a microscopical examination of tissue, made after the death of the patient, is unsatisfactory; hence the advantage of close observation of pathological processes and histological study of tissue excised during life. For it may here be stated that the great advance which dermatology has made in the last half century must be attributed to the grand discoveries made with the microscope, and in general to the improved methods and wonderful results of modern pathological research.

The skin, in its anatomy, resembles mucous membrane, and the sweat glands are the analogues of the Malpighian bodies of the kidneys. Comparative microscopic study shows that the ultimate histological elements of the secretory and excretory organs are epithelial cells and connective tissue, which, in these different organs, are present in varying proportions and peculiar organization or arrangement. On account of this histological fact, it is nothing remarkable that we should be able to recognize in the skin certain general pathological changes that are common to other organs. In the epidermis there may be primary changes due to interference with the nutrition of the cells, it being increased or diminished, in the former case giving rise to hypertrophy, in the latter to atrophy. Characteristic changes may also occur from the presence and growth of certain parasites. It may suffer secondarily through changes that may take place in the corium. The corium contains the glandular system, the blood-vessels, nerves and lymphatics, and it is here that we find primarily the greatest number of morbid changes. To a consideration of these general pathological changes I wish to invite your thought.

Hyperæmia consists of an increased amount of blood in a part, and is due to a dilatation of the blood-vessels. The calibre of the blood-vessels is under the control of a special system of spinal nerves, called the vaso-motor system, in which there are two sets: the *vaso-constrictors*, maintaining the tone of the vessels, and the *vaso-dilators*, operating to cause dilatation. The normal calibre exists as long as a physiological balance is maintained between the action of these two sets of nerves. If the balance is disturbed by irritation of any kind, mechanical, chemical, thermic, etc., causing paralysis of the *vaso-constrictors* or stimulation of the *vaso-*

*dilators*, the calibre of the blood-vessels becomes at once increased in size, which condition permits of the presence of an increased amount of blood. Hyperæmia may be of two kinds, active and passive.

Active hyperæmia is characterized by an increase in the amount of blood in the arteries and capillaries of a part, giving rise to a bright red arterial coloration, and in the skin to an elevation of the temperature of the part. The blood current is at first accelerated, on which account the blood has less time to yield up its oxygen and take up carbonic acid. This causes the bright red color. The acceleration of the current also causes in the skin the elevation of temperature. But the dilatation of the vessels is limited to a certain locality, and after a proper amount of blood has rushed in, the current is slowed, and there is no longer any rise of temperature. These changes are therefore only temporary. Active hyperæmia is the initial stage of another important pathological process—inflammation—and it is impossible to draw a sharp line of demarcation between the former and mild forms of the latter.

Passive hyperæmia, on the other hand, consists of an increased amount of blood in a part, due to obstruction in the venous circulation. The capillaries and venules become distended with blood, and a condition of stagnation ensues. On account of this retardation of the blood current, there results a lowering of temperature when the skin is thus affected, and the blood, becoming rapidly de-oxygenated, assumes a darker hue, giving rise to a diffuse deep red or violaceous coloration. Repeated or chronic hyperæmia of the skin gives rise to more or less pigmentation, as in the case of varicose veins of the lower extremities.

Inflammation is a pathological process which plays an all-important rôle in a large percentage of cutaneous diseases. Yet, notwithstanding the great advances in chemical knowledge and the admirable results attained by late histological inquiry, it is not yet possible to give an exact definition of inflammation—a definition representing the precise nature of the process. The most that can be done is to give a description founded upon the latest and most reliable histological research. It is still customary to give the cardinal symptoms of Galen, such as heat, swelling, redness, pain, with the addition of impaired function, as a definition. This has been and still is, especially in diseases of the skin, useful from a clinical point of view. But recent investigations, especially those of Stricker, show that these symptoms are by no means constant, and are therefore unreliable. Thus pain, associated only with irritation of the sensory nerves, is no symptom of inflammation of the tissues of internal organs, such as lungs and liver, where such nerves are wanting. It seldom occurs in the perifollicular inflammation of *acne* and it is not constant in *psoriasis.*

The position of the skin favors heat radiation, and normally the temperature of its tissues are thus much lower than that of the blood. In inflammation the temperature of the cutaneous tissue is elevated nearly to that of the blood as a result of an acceleration of the blood current and the increased amount of blood in the part. The temperature, however, according to authorities, never reaches that of the blood in the heart, and this corresponds to the observation that there is no rise of temperature in inflammation of internal organs.

Then, again, swelling is not present in *acne rosacea* and is often imperceptible in *eczema erythematosum.* In addition, these cardinal symptoms do not by any means cover the phenomena of chronic inflammation. The symptoms which appear to me reliable, on account of their constancy, and which must take the place of the old cardinal symptoms are, first, *active hyperæmia* (fluxion); second, *active tissue metamorphosis.* These are features which, according to the investigations of Stricker, are characteristic and must exist wherever there is inflammation.

The process of inflammation may be divided into, first, vascular disturbance; second, infiltration; third, resolution or suppuration. Inflammation is always preceded by vascular disturbance, consisting of hyperæmia, which, on the surface of the skin, gives rise to redness and increased heat. Through dilatation of the blood-vessels the walls become thinner and filtration of the blood serum is favored. When the hyperæmia is at its height the blood current is slowed and finally a condition of stasis ensues. A microscopical examination of the exposed mesentery of a frog reveals the lumina of the vessels crowded with corpuscles of which the white corpuscles are seen disposed to cling to the walls of the vessels, and the red corpuscles arranged more in the centre of the vessels. Stasis of the blood favors migration, and both the red and the white corpuscles may be seen to wander out through the walls of the blood-vessels into the surrounding tissue. This exudation from the blood, together with the phenomenon of migration, may accompany every process of inflammation (though this has never been shown), but that it is the essential and characteristic feature, as claimed by Cohnheim and his followers, has not been established. The thorough histological studies of inflammation as it occurs in different tissues, such as the cornea, cartilage, tendon, bone and skin made by Stricker and his school, have shown that during the stage of active hyperæmia the cells composing the tissue swell, causing the induration so well known in acute inflammation, and they acquire the characteristics of embryonic cell life, being capable of amœboid motion and proliferation. This gives rise to the infiltration which may be supplemented by an exudation from the blood. This active tissue metamorphosis occurs to a greater or lesser extent, and the inflammation is in proportion to it. The composition of the infiltration determines the character of the lesion; when composed mostly of serum, it gives rise to lesions like the vesicle or bleb; when mostly cellular in its constituency, there occur lesions after the nature of papules or tubercles; should the infiltration end in suppuration, lesions such as pustules or abscesses are developed.

Inflammation may be acute or chronic. The process is regarded as acute when it goes through the different stages and reaches definite conditions in a certain time, dependent to a great extent upon the amount and character of the irritation. Chronic inflammation is either the issue of an acute condition, or is from the beginning a chronic process. The former case is often illustrated in the tissues of the skin and mucous membranes, while the latter is found to take place in internal organs. In the latter case the process is never followed by suppuration.

Inflammation terminates either in resolution or suppuration. By resolution is understood the absorption of fluids present by the lymphatics and the return of the cells to the normal condition. It is a re-establishment of the integrity of the tissues involved. When there has been much tissue change and the infiltration is largely cellular, the serum may be absorbed and the cells may develop into fixed elements, thus in the corium forming connective tissue. This result is an inflammatory hypertrophy. Finally inflammation may terminate in suppuration. The tissue elements having returned to their embryonic state, proliferate and assume all the characteristics of embryonic cell life. This cell proliferation is necessarily attended with a disintegration of the tissue and the result is the formation of pus. Pus is a fluid mass of more or less consistence, containing pus corpuscles, fatty degenerated cells, granules and cell debris. Suppuration is therefore the issue of an over-production of cells, the result of active tissue metamorphosis, always attended with destruction or loss of tissue which may or may not be permanent. As to the skin, a destruction of the tissue of the corium is permanent, and the loss is replaced by a development of cicatricial connective tissue. In the epidermis the loss is repaired by the development of new epidermis.

Other forms of degeneration of cutaneous tissues may follow inflammation, such as gangrene or complete death of the part. Colloid, amyloid, mucoid and calcarious degeneration are rare, while cheesy degeneration is more common, especially in individuals of a scrofulous or tuberculous habit.

Hemorrhage occurs, as a rule, through rupture of blood-vessel walls, *per rhexin*, whereby the blood flows into the surrounding tissues. It has also been established that the elements of the blood may pass through the intact blood-vessel wall *per diapedesin*. As to the skin, hemorrhage may take place into any of the layers and glands, and the discoloration produced in the tissues varies according to position. In the subcutaneous connective tissue, a hemorrhage is more or less diffuse and presents on the surface a dark bluish tint. In the corium it may occur in spots or streaks, and as in the rete mucosum the discoloration is dark red, later brown. Where the hemorrhagic infiltration is limited, it may be quickly absorbed, but where it is considerable, it requires time for complete disappearance. The corpuscles undergo degeneration, the red corpuscles first, the same soon yielding up their coloring matter, which is quickly absorbed by the surrounding tissues, causing the discoloration, at first brownish red, then green, then yellow. Often a slight pigmentation persists for a time at the seat of a hemorrhage.

Hypertrophy consists of an increase in the amount of tissue composing any organ of the body. This may be due either to an increase in the size of the ultimate histological elements, constituting simple hypertrophy, or to an increase in the number of these elements constituting numerical hypertrophy or hyperplasia. This condition is usually marked and is the result of increased nutrition. It may affect any of the layers or appendages of the skin; thus there may be hypertrophy of the cellular layers, the pigment or the connective tissue of the corium; also of the glands, hair and nails.

Atrophy is a retrograde change in which, from impaired nutrition, the amount of tissue in any organ becomes diminished. The process may consist of a diminution in the size or number of tissue elements, in the former case being called simple, in the latter numerical atrophy. It is a process opposite to that of hypertrophy, but may likewise affect any part of the skin or appendages and may be general or partial.

Tumors, or new formations, are pathological products resulting from a non-inflammatory development of tissue. They may occur in any part of the organism, in any tissue, and differ from one another in their anatomical, etiological and clinical relations. These products have been variously classified, but the most scientific classification is the one proposed by Virchow, having a histogenetic basis. Clinically they are regarded as benign or malignant, the former exercising as a rule no harmful influence on the surrounding tissue, while the latter have in them potentially those properties which tend to destroy life. The most dangerous of these properties is that of metastasis, or dissemination of the growth through the lymphatics or blood-vessels to other organs. There is, however, no sharp line of division between the two forms, as an apparently benign tumor sometimes develops into a malignant one. Tumors of the skin form a distinct group, and the development may proceed from the cellular layers of the epidermis or glandular apparatus, forming *papilloma* and *epithelioma*—from the connective tissue of the corium and subcutaneous layers, forming *fibroma* and *lipoma*—or from the blood-vessels, *angioma*—from the lymphatics, *lymphangioma*—from the nerves, *neuroma*.

Parasites are organisms, vegetal or animal, whose habitat and means of subsistence are maintained at the expense of some other organism. All the structures of the human body are liable to be invaded, but more especially those that are exposed and are brought into intimate relations with the external world, as the skin and mucous membrane.

Parasites may infest the cutaneous tissues and provoke hyperæmia, inflammation, hemorrhage, desquamation in varying degrees according to the nature of the parasite. They are divided into vegetal and animal.

The vegetal parasites belong to the low order of plants called fungi, and of these, three kinds have been recognized as having relations with certain cutaneous diseases. The *achorion schonleinii*, discovered by Schönlein, is found in *favus*. The *trichophyton*, discovered by Malmsten, is associated with *tinea tonsurans*, or ring-worm of the scalp, *tinea circinata*, or ring-worm of the body, and *tinea sycosis*, or barber's itch. The third species is the *microsporon furfur* of Robin. These fungi grow only in the epidermal structures, especially in the horny layers, and when examined microscopically are found to consist of fine threads called *mycelium*, and masses of minute bead-like bodies called *spores* or *conidia*. Their predilection for certain localities is worthy of notice, as *tinea favosa* for the head, *microsporon furfur* for the chest and back. A proper soil is requisite for the development of fungi. This perhaps explains the fact that, although the diseases to which they give rise are regarded as contagious, every individual does not equally offer the requisite conditions for their growth, and that they do not flourish as readily on some as on others.

The animal parasites that may invade the skin belong to the class called arthropods. The most common are the *acarus or sarcoptes scabei* giving rise to *scabies*, or itch, and the *pediculus*, or louse, producing characteristic hemorrhagic lesions. Besides these, there are other species of insects that attack the exposed parts of the skin, but do not make the skin their habitat, such as the *climex lectularius*, or bed-bug, *pulex irritans*, or flea, *culex pipiens* or mosquito.

